# A Case of a 34-Year-Old Female with Acute Hypoxemic Respiratory Failure and Proximal Muscle Weakness

**DOI:** 10.1155/2017/7941715

**Published:** 2017-12-19

**Authors:** Alex Diaz, Surit Sharma

**Affiliations:** Sky Ridge Medical Center, Denver, CO, USA

## Abstract

Wound associated botulism is an unusual presentation. Early detection of this potentially life-threatening illness can significantly shorten length of hospital stay and improve prognosis. We present a case of a 34-year-old female with a history of heroin abuse who presented to the ED with acute respiratory failure, diplopia, and proximal muscle weakness. There was early concern for wound botulism as the instigating process. After discussion with the CDC, she was given equine serum heptavalent botulism antitoxin. Laboratory analysis later confirmed our suspicion. Symptoms improved and the patient was liberated from mechanical ventilation on day 14 and discharged from the hospital on day 23.

## 1. Case

This is a case of a 34-year-old female with history of heroin abuse by “skin-popping,” pseudosyncope, and current incarceration who presented to an Emergency Department for progressive dyspnea.

She was seen at a different hospital two days priorly with complaints of throat tightness. She was treated with cephalexin and trimethoprim-sulfamethoxazole for possible pharyngitis and then discharged back to county jail. She later developed dyspnea, diplopia, and proximal muscle weakness.

It was assumed that the patient may have been having a reaction to one of the two antibiotics. Later that day, she was found less responsive leading to ambulance transfer to the Emergency Department. En route, she was noted to be hypoventilating and hypoxemic requiring bag-valve-mask-ventilation.

Upon arrival to the Emergency Department, she was intubated urgently and placed on mechanical ventilation. At the time of intubation, she was noted to be “drooling.” She was given naloxone without benefit. A lumbar puncture was performed and she was admitted to the medical ICU.

Physical exam at that time showed blood pressure being 190/100, temperature 96.8°F, pulse 118 beats/minute, and 16 respirations per minute on mechanical ventilation with a FiO2 of 60%. Her pupils were sluggishly reactive to light. Her facial and extraocular muscles were weak. She had an absent gag reflex and was unable to open her eyes and lift her head, shoulder, or hips. She had 5/5 distal muscle strength. The patient was appropriately responsive to questioning with thumb hand signals and then later by writing. She had absent patellar and biceps tendon reflexes and an absent Babinski reflex. Gross sensation was intact. Her skin had multiple track marks. She had a 1.5 cm lesion under her right breast without exudate. She also had two lesions on her right hip that were about 2 and 3 cm wide without exudate as well.

Complete blood count indices on admission revealed white blood cells count of 18.3, hemoglobin of 13.0, hematocrit of 42.6, and platelets of 533. Her sodium, potassium, chloride, carbon dioxide, BUN, creatinine, glucose, and calcium were all within normal limits. TSH was 5.570. ABG prior to intubation had a pH of 7.26, pCO2 46.9, pO2 200, and HCO3 21.0. Urine toxicology screen was positive for only opioids. Portable AP chest X-ray showed no infiltrates, pneumothorax, or effusions. CT of the head and neck without contrast demonstrated a hypoplastic thyroid but no other acute abnormalities.

At the time of admission, diagnoses of wound botulism, drug overdose, tetanus, meningoencephalitis, Guillen-Barre/Miller-Fisher syndrome, myasthenia gravis, and Lambert-Eaton syndrome were entertained. Blood samples were sent to CDC headquarters in Atlanta for analysis, and equine serum heptavalent botulism antitoxin was procured for administration and given empirically. She was also started empirically on metronidazole and vancomycin for* Clostridium botulinum* and gram-positive cocci. Clindamycin was started to blunt toxin production. Antibiotics were later changed to single agent piperacillin-tazobactam once wound cultures demonstrated methicillin-sensitive* Staphylococcus aureus* and coagulase-negative staphylococci. She was treated for a total of two weeks. No anaerobic organisms were ever cultured despite multiple samples.

Initial laboratory results revealed a CSF cell count of 1 WBC/ul and 2 RBC/ul, a glucose level of 66 mg/dl, and a protein level of 39 mg/dl. CSF cultures were negative. Lambert-Eaton radioimmunoassay for anti-VGCC antibodies was negative. GQ1b antibody titer for Miller-Fisher syndrome was <1 : 100. Antimuscarinic and acetylcholine receptor blocking, binding, and modulating antibodies were also all negative. Eventually, on ICU day 12, the CDC's sample analysis demonstrated serum botulism toxin confirming the diagnosis of wound botulism.

Patient's hospital course was characterized by slow improvement of motor function. After failing an attempt at extubation, a percutaneous tracheostomy and a percutaneous gastrostomy tube were placed. By day 14, she was liberated from mechanical ventilation but tracheostomy was kept in place to help airway clearance given a weak cough. By day 23 (the day of transfer to a long-term acute care hospital), she had regained facial and proximal muscle strength but still demonstrated mild difficulties in swallowing.

## 2. Discussion

Wound botulism is a rare condition. This case illustrates that early recognition and empiric treatment can significantly impact length of stay, mortality, and hospital cost.

According to the CDC, there were a total of 161 confirmed cases of botulism in the United States in 2014. Of that number, only 16 cases were wound botulism. All documented cases were in injection drug users. All cases were reported from either California or Texas [1].

A retrospective, case-control study of 136 patients by Passaro et al. revealed that wound botulism occurs almost exclusively among drug users whose route is either subcutaneous or intramuscular as compared to intravascular route. They also demonstrated that the infection rates were dose-dependent with higher doses leading to increased rates of wound botulism. 26 of the patients in the study were intravenous drug users who developed wound botulism from 1994 to 1996 and the control group consisted of 110 intravenous drug users who recently enrolled in a methadone detoxification program. Patients were more likely than controls to inject drugs subcutaneously or intramuscularly (92% versus 44%, *P* < .001) and used this route of drug administration more times per month (mean, 67 versus 24, *P* < .001), with a greater cumulative monthly dose of black tar heroin (22.3 g versus 6.3 g, *P* < .001) [2]. With our case, the patient stated that she did heroin frequently and this was also evident by numerous skin lesions. “Skin-popping” or depositing the drug subcutaneously was our patient's route of choice as well for deliverance of heroin.

Making the diagnosis of wound botulism is a difficult but important task. Most of the time, clinicians must rely on history and physical examination. Wound botulism presents in a manner similar to food-borne botulism but lacks gastrointestinal symptoms and fever and leukocytosis are often more common. Incubation is usually longer and may take up to two weeks before symptoms develop [3]. For our patient, she admitted to last heroin use being nine days prior to the start of her symptoms. Wheeler et al. have estimated a serum toxin assay sensitivity of 68% [4]. Another useful diagnostic modality is electromyography (EMG) and RNS (repetitive nerve stimulation). Many case reports cite using EMG and/or RNS as a helpful tool in identifying wound botulism early. Testing should be done between 20 and 50 hz until 4–10 compound muscle action potential amplitudes are seen for RNS. Results for botulism usually show a 100% increase from first to last action potential on repetitive nerve stimulation testing and greater than 10% fiber potentials exceed normal jitter or impulse blockade on electromyography [5, 6]. For our patient, we were unable to perform these tests due to unavailability at the hospital.

Hospital and ICU length of stay is another critical aspect of wound botulism. Increased ICU length of stay has been attributed to increased morbidity, mortality, and cost. As stated priorly, early diagnosis is a key factor in decreasing hospital and ICU length of stay. A retrospective review done by Offerman and colleagues confirmed a correlation between time of presentation and antitoxin administration with ICU length of stay. Their study demonstrated a regression coefficient of 2.5 with a 95% CI of 0.45–4.5. 18 of the 29 patients who met inclusion criteria of being an intravenous drug user with confirmation of wound botulism received antitoxin by 24 hours. Of these patients, all except five had a hospital length of stay less than 30 days with three more having an unknown duration of stay. The mean length of hospital stay for patients receiving antitoxin by 24 hours was 26.2 days. All 18 patients receiving antitoxin by 24 hours had an ICU stay less than 30 days. Only one patient's ICU course was unknown. Mean ICU length of stay for these patients was 13.7 days [7]. Even though our patient was not at a facility that stocked botulism antitoxin, we were still able to give the antitoxin within 24 hours of presentation. Based on published observations, we believe that our patient's hospital length of stay correlates with early antitoxin administration. Pittet et al. did a case-control study of SICU patients comparing development of a nosocomial infection with ICU length of stay, mortality, and cost. Their results showed that hospital length of stay (40 days versus 26 days, *P* < .01) and mortality (50% versus 15%, *P* < .01) were greater in the case compared to the control groups. These differences were attributed to an average increase per surviving patient of 40,000 dollars [8].

## 3. Conclusion

Wound botulism is a rare and difficult diagnosis to make. It is imperative to have a high clinical suspicion in any intravenous drug user who presents with dyspnea, proximal muscle weakness, diplopia, and skin lesions. Early suspicion and prompt treatment with empiric antitoxin are critical to decrease mortality, length of stay, and hospital cost. Wound botulism can be easily misdiagnosed and this may lead significantly to worse outcomes.
